# Synthetic polarization-sensitive optical coherence tomography by deep learning

**DOI:** 10.1038/s41746-021-00475-8

**Published:** 2021-07-01

**Authors:** Yi Sun, Jianfeng Wang, Jindou Shi, Stephen A. Boppart

**Affiliations:** 1 Beckman Institute for Advanced Science and Technology, University of Illinois at Urbana-Champaign, Urbana, IL USA; 2 Department of Electrical and Computer Engineering, University of Illinois at Urbana-Champaign, Urbana, IL USA; 3 Department of Bioengineering, University of Illinois at Urbana-Champaign, Urbana, IL USA; 4 Carle Illinois College of Medicine, University of Illinois at Urbana-Champaign, Urbana, IL USA; 5 Cancer Center at Illinois, University of Illinois at Urbana-Champaign, Urbana, IL USA

**Keywords:** Biophotonics, Cancer imaging, Imaging and sensing, Computational science

## Abstract

Polarization-sensitive optical coherence tomography (PS-OCT) is a high-resolution label-free optical biomedical imaging modality that is sensitive to the microstructural architecture in tissue that gives rise to form birefringence, such as collagen or muscle fibers. To enable polarization sensitivity in an OCT system, however, requires additional hardware and complexity. We developed a deep-learning method to synthesize PS-OCT images by training a generative adversarial network (GAN) on OCT intensity and PS-OCT images. The synthesis accuracy was first evaluated by the structural similarity index (SSIM) between the synthetic and real PS-OCT images. Furthermore, the effectiveness of the computational PS-OCT images was validated by separately training two image classifiers using the real and synthetic PS-OCT images for cancer/normal classification. The similar classification results of the two trained classifiers demonstrate that the predicted PS-OCT images can be potentially used interchangeably in cancer diagnosis applications. In addition, we applied the trained GAN models on OCT images collected from a separate OCT imaging system, and the synthetic PS-OCT images correlate well with the real PS-OCT image collected from the same sample sites using the PS-OCT imaging system. This computational PS-OCT imaging method has the potential to reduce the cost, complexity, and need for hardware-based PS-OCT imaging systems.

## Introduction

Polarization-sensitive OCT (PS-OCT)^1^ has become an exceptionally useful imaging technique that complements the scattering intensity contrast in standard OCT systems and provides additional diagnostic information and guidance^2–7^. By analyzing the polarizations of the scattered light, PS-OCT imaging systems measure the birefringence within the imaged tissue sites, which is largely related to the orientation and organization of tissue structures. For example, collagen fibers aligned in a dominant direction display a high birefringence compared to randomly aligned collagen fibers^8^. It has also been shown that the additional polarization information provided by PS-OCT facilitates the visualization of unrecognizable features in OCT images^4,9,10^. In particular, PS-OCT has been used in clinical applications^2,6–8^ to effectively differentiate cancer tissue and connective tissue based on their different birefringence, while these types of tissues appear very similar in standard OCT images.

While today’s standard OCT imaging system can fit into a briefcase^11^, most PS-OCT imaging systems require the use of multiple detectors as well as many polarization-manipulating optical components, which increase the cost and complexity of the imaging system^2–5^. Handheld probe integration is another major issue for PS-OCT systems because the bending of the fiber-optic delivery cable can often alter the polarization state in unpredictable ways, and introduce artifacts in the images. The intraoperative use of OCT has been widely explored using handheld probes so that surgeons can collect OCT images in real-time to assess tumor margins and lymph nodes in cancer patients^8,12,13^. Intraoperative probe-based PS-OCT imaging can generate images based on the presence and organization of collagen structures in normal breast tissues, as well as on the absence or derangement of these structures in cancer^2,8^. However, the widespread use of handheld PS-OCT may be limited because of the added complexity associated with these PS-OCT systems.

Conventional PS-OCT imaging systems typically record multiple OCT images with different polarization states to compute the two major PS-OCT contrast metrics, the phase retardation and the degree of polarization uniformity (DOPU). However, based on our experience with PS-OCT and OCT images of different tissues, we believe that the birefringence is related to the reflectance within the tissue. Therefore, the polarization information should be fundamentally embedded in the OCT intensity image collected along one polarization and can be potentially extracted via computational methods. In this study, we propose and demonstrate the use of deep learning to synthesize polarization-sensitive contrasts with single-polarization OCT intensity images, eliminating the extra cost (thousands of dollars) for multiple detectors and polarization optics and fiber components used in conventional hardware-based PS-OCT systems.

Over the past few years, deep learning has been extensively applied in image contrast translation tasks^14–21^. For instance, a U-Net is a commonly used deep neural network (DNN) for biomedical image segmentation^22,23^ which converts the original image contrast into a binary feature mask. However, when it comes to generating complex image contrasts instead of only binary masks, the performance of a U-Net is often compromised unless large training datasets are used^14,18^. In response to this, the generative adversarial network (GAN)^15,24^ was adopted to enable more accurate image translation with small datasets. A GAN has its unique structure by incorporating a generator network and a discriminator network^24^. While the generator network is similar to the DNNs used in conventional feature segmentation like U-Net, the discriminator network scores the synthetic images to estimate how likely they come from the training dataset instead of the generator network. With such a unique network structure, a GAN was used in various synthetic biomedical imaging applications, such as virtual histology^15^, digital phase staining^17^^,^ and synthetic clinical imaging^20,25^. Based on these demonstrated results, we propose and demonstrate the use of a GAN as the deep-learning model to synthesize PS-OCT images.

## Results

### Synthetic PS-OCT imaging by GAN

**Fig. 1:**
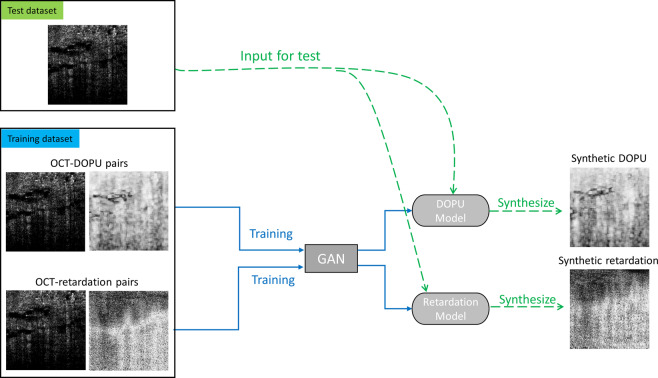
Workflow of computational PS-OCT imaging. Two models were separately trained to synthesize DOPU and phase retardation contrasts with OCT intensity images as the input.

**Fig. 2:**
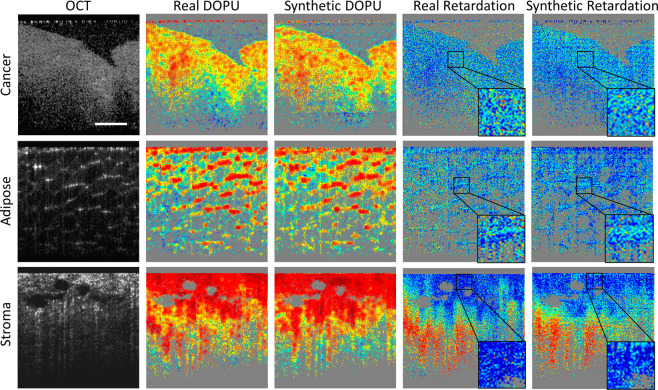
Representative computational PS-OCT images from cancer, adipose, and stroma tissue specimens, compared with the real PS-OCT images. The noisy areas within the phase retardation images are enlarged to directly compare. The 200 μm scale bar applies to all images.

The framework of the training and testing process is shown in Fig. 1. The collected OCT and PS-OCT images were combined as pairs to train the GAN. After training, two GAN models were generated for the DOPU and phase retardation synthesis, two common image-based representations of polarization information in PS-OCT images. As shown in Fig. 2, the synthetic contrasts of DOPU and phase retardation were overlaid with the “jet” color map on the OCT intensity images that were processed by zeroing the pixel values under a specified threshold. Figure 2 shows representative OCT and PS-OCT images from different types of human breast tissues in the test datasets, including adipose tissue, stromal tissue, and tumor tissue. The real and synthetic PS-OCT images look visually similar to one another. Furthermore, to quantitively evaluate the quality of contrast synthesis, we calculated the structural similarity index (SSIM) between the real and synthetic images^26^. After omitting the image pixels under the intensity threshold, the SSIM values of images were calculated to be 0.8531 ± 0.0699 for the DOPU contrast in the test datasets, and 0.6659 ± 0.0517 for the phase retardation contrast. It is noticed that the SSIMs of the phase retardation images are relatively low in contrast synthesis tasks. This is probably because the phase retardation images intrinsically have a higher level of noise than the DOPU images, and therefore, a lower signal-to-noise ratio (SNR)^27,28^. Due to the low SNR and the random nature of the noise, the GAN struggled in learning and precisely synthesizing the noise pattern in the phase retardation images. As shown in the enlarged phase retardation images (black boxes) in Fig. 2, despite the overall similar image features, the local noise patterns are very different between the synthetic and real phase retardation images.

### Validation by deep-learning-based image classification

**Fig. 3:**
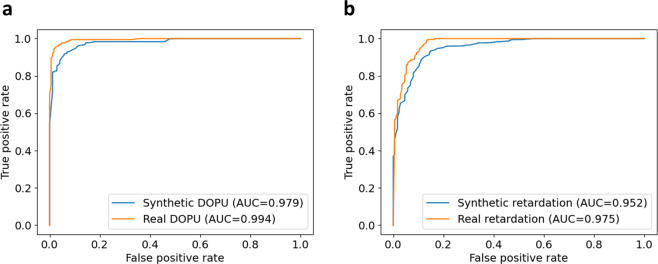
ROC curves of cancer/normal classification using real and synthetic PS-OCT images. Classification results trained on (**a**) DOPU and (**b**) phase retardation contrasts. The ROC curves of the synthetic images are largely overlapping with the curves of the real images, and the AUC of the two curves are also similar.

In addition to evaluating the synthesis quality by SSIM, we also examined the effectiveness of using the synthetic PS-OCT images instead of the real PS-OCT images in cancer/normal classification^29,30^. The real and synthetic PS-OCT images in the test datasets from the GAN were used to train a classification DNN based on ResNet-18^31^ that generated two classifiers to differentiate images collected from cancer or normal tissues. The similarity between the classification results of these two classifiers can be used to demonstrate the effectiveness of using synthetic PS-OCT images for cancer/normal classification. To evaluate the classification accuracy, the receiver operating characteristic (ROC) curves of the two classifiers are shown in Fig. 3. In Fig. 3a, the ROC curves from synthetic DOPU datasets are in close proximity with the real DOPU datasets. In addition, the area under curves (AUCs) of both ROC curves in Fig. 3a approach the ideal value of one (0.979 for synthetic DOPU, and 0.994 for real DOPU). Observing the ROC curves of the phase retardation datasets in Fig. 3b, the AUCs of the two curves are slightly lower than the DOPU classifiers (0.952 for synthetic phase retardation, and 0.975 for real phase retardation), but the two ROC curves from the real and synthetic datasets are also largely overlapping. The equally good performance of the classifiers indicates that the synthetic PS-OCT images can likely be used interchangeably with the real PS-OCT images for cancer/normal classification. From the AUC results, we noticed that the difference between the real and synthetic datasets is 0.015 for DOPU images, 0.007 smaller than 0.023 for phase retardation images. Compared to the relatively large SSIM difference between the DOPU (0.8531 ± 0.0699) and phase retardation (0.6659 ± 0.0517) images, the difference in AUC performance (0.007) is negligible. As explained in the previous section, the low SSIMs of phase retardation images can be attributed to the poor prediction of the noise pattern. In addition, the ResNet-18 classifier can pick up the useful tissue feature information from the noise. Therefore, the difference in the noise pattern has little effect on the classification results, and it is the well-predicted overall tissue features that contribute to the good classification performance. This finding indicates that the synthetic phase retardation images, even with low SSIM, are qualified for various PS-OCT image processing applications.

**Fig. 4:**
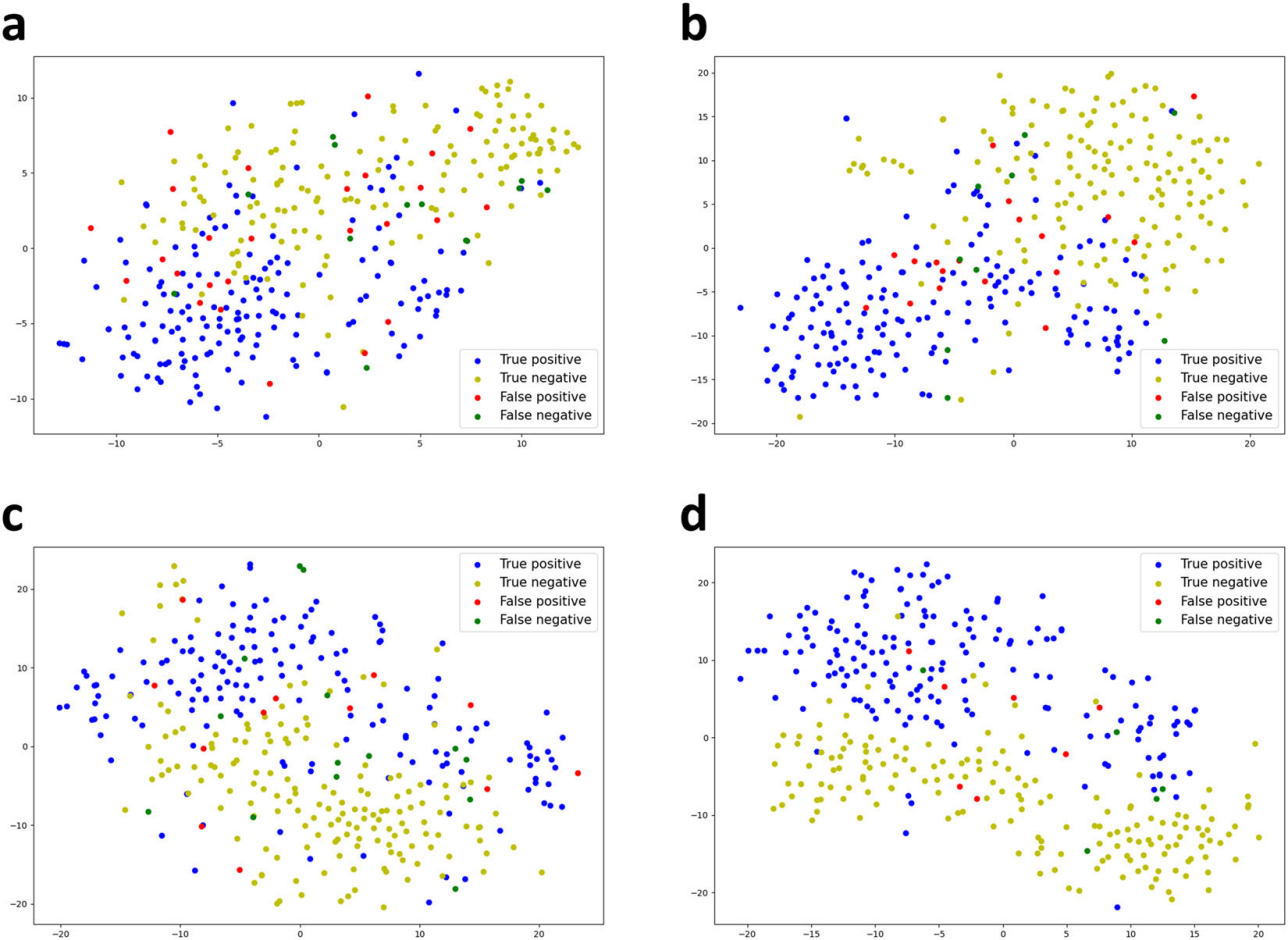
t-SNE representation of the last activation map in the classification models. The t-SNE data distribution is included for (**a**) synthetic retardation, (**b**) real retardation, (**c**) synthetic DOPU, and (d) real DOPU images. The distribution of the classified synthetic PS-OCT images resembles the distribution of the real PS-OCT images.

In addition, to visualize the distribution of the classified real and synthesis images as two-dimensional data points, we ran t-SNE analysis on the last activation map generated in the classifier network^32^. The data distribution visualized in Fig. 4 is used to study the relationship of the synthetic and real PS-OCT images in the image vector space. Each data point in Fig. 4 represents one PS-OCT image in the test datasets, and each is colored by the classification result. The scatter map of the colored data points shows how these PS-OCT images are distributed in their transformed vector space. As shown in Fig. 4a and b, the distribution of the synthetic DOPU images is similar to the distribution of the real DOPU images, in which the true positive data points are located in the lower-left region of the map. The t-SNE results of the classified phase retardation images are shown in Fig. 4c and d, and the distributions of the real and synthetic phase retardation images are also similar.

### Testing on OCT images from a separate OCT-only system

**Fig. 5:**
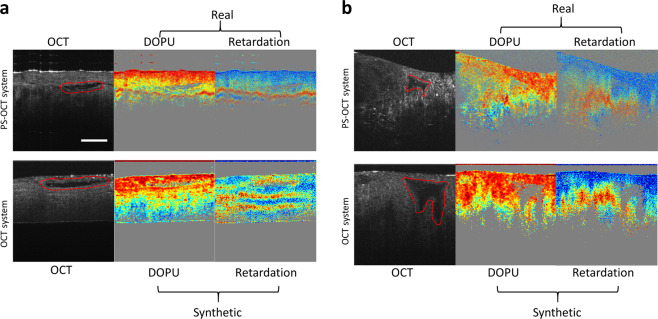
Application of the prediction models on images collected by a standard OCT-only imaging system with the same wavelengths as the PS-OCT imaging system. By imaging the same tissue sites from (**a**) chicken skin and (**b**) chicken muscle, the synthetic PS-OCT images from the OCT system were directly compared with the real PS-OCT images collected by the PS-OCT system. Similar features are encircled by the red lines. The 200 μm scale bar applies to all images.

The ability of the GAN model for contrast synthesis was further tested on OCT images collected from a separate standard OCT imaging system that did not have any PS-OCT hardware or processing components. We imaged fresh chicken tissues with both the PS-OCT system and a standard OCT system that used similar imaging wavelengths. The model was re-trained on the PS-OCT images of chicken tissues and applied to the OCT images from the OCT system. In Fig. 5, the synthetic PS-OCT images from the OCT system were compared with the real PS-OCT images. For image correlation, two unique structures (red lines in Fig. 5) are identified in images collected from the OCT and PS-OCT systems, confirming that the two imaging systems collected images from the same tissue sites. Figure 5a shows the real and synthetic PS-OCT images of chicken skin tissue, while Fig. 5b shows the images collected from chicken muscle tissue. The trained GAN models of DOPU and phase retardation gave reasonable synthetic contrasts on the images collected by the OCT system. Despite some local discrepancies due to the challenge of imaging the same location with two different systems with micron-level precision, the real and synthetic PS-OCT images correlate well on a larger scale.

## Discussion

We developed a computational method to synthesize PS-OCT images by deep learning, aiming to eliminate the need for multiple detectors and polarization optics in conventional PS-OCT imaging systems. While conventional hardware-based PS-OCT requires at least two OCT images with different polarizations to obtain the DOPU and phase retardation contrasts, our method managed to extract the polarization-sensitive information from single-polarization intensity images from a standard OCT system. The computed SSIMs of the synthetic PS-OCT images are reasonably high to accurately represent the real PS-OCT images, especially for the DOPU contrast. Besides the mathematical similarity between the synthetic and the real images, we were also particularly curious about how these synthetic images perform in common applications of PS-OCT imaging and whether they can substitute the real images in these applications to achieve similar results. The promising results of the classification, including the largely overlapping ROC curves and comparable AUCs, confirmed that the synthetic PS-OCT images serve equally well as the real PS-OCT images in cancer/normal classification applications. Furthermore, t-SNE analysis showed that the classified synthetic PS-OCT images distribute in their vector space in a similar way as the real PS-OCT images. Therefore, the similarities between the real and synthetic PS-OCT images were examined not only by SSIM, but also in the perspective of deep-learning-based applications of PS-OCT. Finally, effective PS-OCT synthesis with OCT images from a separate OCT-only system signifies the robustness of this model and the potential for applying it on images collected from different types of OCT systems, such as the ultra-compact and economical OCT system that can fit into a briefcase^11^.

It has been demonstrated in this study that the GAN model trained by OCT and PS-OCT images can synthesize PS-OCT images that can be used in cancer/normal classification. However, there are still several issues in our work to explain further. First, the average 0.6659 SSIM of the synthetic phase retardation images is relatively low among deep-learning-based contrast synthesis studies^15–17^. For instance, the work of MRI image synthesis gave an SSIM ranging from 0.877 to 0.926^33^. It has been explained that the phase retardation images have a significantly higher level of noise, and subsequently, a lower SNR. We expect this issue to be overcome by applying noise-suppression methods in the calculation of the Jones-matrix, such as the adaptive Jones matrix averaging method^27^. With a lower level of noise, the quality of synthesis and the SSIM should be greatly improved. The second issue lies in the correlation between the real PS-OCT images and the synthetic PS-OCT images generated from another OCT system. With the tissue being inevitably perturbed and distorted when transferred from one imaging system to another, it becomes impractical to directly compare on the micron scale the synthetic PS-OCT images with the real ones. To demonstrate the robustness of the GAN model, we need another PS-OCT system to provide the co-registered PS-OCT images that work as the ground truth. Furthermore, to improve the robustness of the model and make it more universal, the GAN should be trained by images collected from many different kinds of PS-OCT systems.

In conclusion, synthetic PS-OCT imaging by deep learning was demonstrated to address many of the common limitations of current PS-OCT imaging. The quality of synthetic PS-OCT imaging was evaluated by conventional image quality metrics SSIM as well as the outcome of cancer/normal classification. The evaluation results demonstrated that the synthetic PS-OCT images can potentially serve as substitutes for real PS-OCT images. Finally, the use of this PS-OCT synthesis model on images from an OCT-only system suggests that synthetic PS-OCT imaging can replace hardware-based PS-OCT imaging systems to reduce cost and complexity and to potentially resolve the problems of artifacts associated with handheld fiber-delivered probe-based PS-OCT imaging.

## Methods

### Image acquisition

The PS-OCT images for training and testing the GAN model were acquired from fresh human breast tissue specimens with a portable PS-OCT imaging system^2^. A total of 22,072 PS-OCT images (256 × 512 pixels) were collected from seven subjects with breast cancer and four normal subjects undergoing breast reduction surgery with no history of breast cancer. Each PS-OCT image includes the OCT intensity, DOPU, and phase retardation contrasts. Human tissue specimens were obtained from subjects at Carle Foundation Hospital, Urbana, IL, who preoperatively provided written informed consent permitting the investigational use of their tissue. Imaging of these specimens was conducted under a protocol approved by the Institutional Review Boards at the University of Illinois at Urbana-Champaign and Carle Foundation Hospital, Urbana, IL. Additional human breast tissue specimens were obtained from the Cooperative Human Tissue Network (CHTN), established in 1987 by the National Cancer Institute in response to an increase in the demand for high-quality biospecimens for cancer research.

### GAN training

Each raw image was first cropped to remove the dark margins and separated into two square-shaped images (each 202 × 202 pixels) that better fit into the GAN network. Then, the original 16-bit images were converted into 8-bit to meet the network requirement. The entire image dataset, composed of 44,144 images, was divided into the training, test, and validation datasets under the ratio of 8:1:1. Because the total number of images was sufficiently large, a smaller number of images were assigned for the test dataset. Here we chose to split the dataset on the image basis to ensure that there are a sufficient number of patients/cases in the test dataset for subsequent image classification. The case-based data splitting strategy was also investigated by including six cancer cases and four normal cases in the training datasets and equally splitting the remaining one cancer and one normal case into the test and validation datasets. The SSIMs of the synthetic PS-OCT images under the case-based splitting strategy is 0.65 ± 0.049 for the phase retardation images, and 0.87 ± 0.53 for the DOPU images, very close to the SSIMs under the image-based data spitting strategy. Therefore, our method of using a GAN for PS-OCT synthesis is robust against the case variances.

The GAN used in this study was modified from the pix2pix GAN^14^ by adding the SSIM into the loss function. In doing so, the loss function of the modified GAN was composed of the discriminator loss, the L1 distance, and the SSIM. The modified loss function helps to improve the SSIM of the synthetic images by at least 20% under the same training conditions. We chose the U-Net as the generator network, and a three-layer discriminator network was used to evaluate the synthetic PS-OCT image generated by the U-Net generator. The selection of the generator and discriminator network is based on the experiments using different networks. In particular, the deeper discriminator networks with more layers did not improve the synthesis quality, but rather delayed the convergence. Therefore, we chose the simple three-layer discriminator network. For the training process, we used an Adam optimizer with a learning rate of 2 × 10^−5^ and a batch size of 1. The learning rate of 2 × 10^−5^ ensures a quick and optimal convergence, and the batch size of 1 is set under the instance normalization. Training of the network was run on a Linux machine (Ubuntu 16.04) with GPU acceleration (Nvidia GTX TITAN). With the current data size, 50 epochs of training required about 16 h.

### Classification of computational PS-OCT images by deep learning

The synthetic and real PS-OCT images in the test dataset of the GAN were used to train an image classifier based on ResNet-18^31^. The test dataset of the contrast-predicting GAN (4,414 images) was further separated into the training, test, and validation datasets for the classifier network with the ratio of 6:3:1. In each dataset of the classifier, all the images were labeled by their categories, which were either cancer or normal. To adapt to the number of classes in this study, the dimensions of the final fully connected layer were changed from 512 × 1000 to 512 × 2. To shorten the training time and improve classification accuracy, we adopted the concept of transfer learning^34^ by initializing the network using pretrained weights that were obtained by training on images from ImageNet^35^. After initialization, all the weights in the classifier network except for the last fully connected layer were fixed during the training process. By doing so, the extraction of spatial features by the pretrained network were retained, while the training process of the PS-OCT images only modified the last fully connected layer that determined how the extracted spatial feature was incorporated into the calculation of the final score of each class. This way, the training time is reduced, and the classification accuracy is higher than the trained network with all weights updatable (AUC~0.85). The classifier was trained for 24 epochs, and the model giving the best accuracy in the validation dataset was used for classification. The trained classifier was applied to the test datasets with 350 images to generate the ROC curve. The calculation of the ROC curve and corresponding AUC was implemented using Python codes.

### Visualization of classified data by t-SNE

Similar to the generation of the output score array by the final fully connected layer, all the layers in the classifier network produce an activation map that is passed to the next layer. The last activation map generated by the second-to-last layer in the network is a 512-elements array. This array was extracted from the network to represent the image with a compressed size. t-SNE analysis was performed on these arrays under standard Euclidean distance, a perplexity of 30, and a random initialization of embedding^32^.

### Reporting summary

Further information on research design is available in the Nature Research Reporting Summary linked to this article.

## Supplementary information

Reporting Summary

## Data Availability

The data that support the findings of this study are available from the corresponding author (S.A.B.) upon reasonable request and through collaborative investigations.

## Code availability

The Python codes that were used for the GAN model, classifier, and t-SNE analysis, as well as the MATLAB codes for data pre-processing and results evaluation in this manuscript are available from the corresponding author (S.A.B.) upon reasonable request and through collaborative investigations.
