# Supplementary material for: Synthetic polarization-sensitive optical coherence tomography by deep learning
Source: NPJ Digit Med. 2021 Jul 1;4:105. doi: 10.1038/s41746-021-00475-8 (PMC8249385; doi:10.1038/s41746-021-00475-8)
Supplement: Supplementary file 1 — Reporting Summary [file 41746_2021_475_MOESM1_ESM.pdf]

nature research

Corresponding author(s): Stephen A. Boppart

Last updated by author(s): Jun 3, 2021

Reporting Summary

Nature Research wishes to improve the reproducibility of the work that we publish. This form provides structure for consistency and transparency in reporting. For further information on Nature Research policies, see our Editorial Policies and the Editorial Policy Checklist.

Statistics

For all statistical analyses, confirm that the following items are present in the figure legend, table legend, main text, or Methods section.

n/a	Confirmed	
☐	☒	The exact sample size (n) for each experimental group/condition, given as a discrete number and unit of measurement
☐	☒	A statement on whether measurements were taken from distinct samples or whether the same sample was measured repeatedly
☐	☒	The statistical test(s) used AND whether they are one- or two-sided<br>*Only common tests should be described solely by name; describe more complex techniques in the Methods section.*
☒	☐	A description of all covariates tested
☒	☐	A description of any assumptions or corrections, such as tests of normality and adjustment for multiple comparisons
☐	☒	A full description of the statistical parameters including central tendency (e.g. means) or other basic estimates (e.g. regression coefficient) AND variation (e.g. standard deviation) or associated estimates of uncertainty (e.g. confidence intervals)
☒	☐	For null hypothesis testing, the test statistic (e.g. F, t, r) with confidence intervals, effect sizes, degrees of freedom and P value noted<br>*Give P values as exact values whenever suitable.*
☒	☐	For Bayesian analysis, information on the choice of priors and Markov chain Monte Carlo settings
☒	☐	For hierarchical and complex designs, identification of the appropriate level for tests and full reporting of outcomes
☒	☐	Estimates of effect sizes (e.g. Cohen's d, Pearson's r), indicating how they were calculated

Our web collection on statistics for biologists contains articles on many of the points above.

Software and code

Policy information about availability of computer code

Data collection	LabView2015 and Thorlab OCT software is used for data collection in this study.
Data analysis	MATLAB2019a,2019b, 2020a, Python 3.6, Pycharm is used for data analysis in this study.

For manuscripts utilizing custom algorithms or software that are central to the research but not yet described in published literature, software must be made available to editors and reviewers. We strongly encourage code deposition in a community repository (e.g. GitHub). See the Nature Research guidelines for submitting code & software for further information.

Data

Policy information about availability of data

All manuscripts must include a data availability statement. This statement should provide the following information, where applicable:

- Accession codes, unique identifiers, or web links for publicly available datasets
- A list of figures that have associated raw data
- A description of any restrictions on data availability

The data that support the findings of this study are available from the corresponding author (S.A.B.) upon reasonable request and through collaborative investigations. Figure 2 and Figure 5 are directly associated with raw data.

Field-specific reporting

Please select the one below that is the best fit for your research. If you are not sure, read the appropriate sections before making your selection.

☒ Life sciences ☐ Behavioural & social sciences ☐ Ecological, evolutionary & environmental sciences

For a reference copy of the document with all sections, see nature.com/documents/nr-reporting-summary-flat.pdf

Life sciences study design

All studies must disclose on these points even when the disclosure is negative.

Sample size	Our study included 22072 images with 256×512 pixels from 11 human subjects.
Data exclusions	No data were excluded from the analyses.
Replication	Multiple deep learning models were generated from separate training processes, and their performance is similar.
Randomization	The images were randomly partitioned into three datasets for deep learning using a random number array.
Blinding	As stated in the randomization, the data allocation were executed by computer-generated random number array, so the investigators were blind to it.

Reporting for specific materials, systems and methods

We require information from authors about some types of materials, experimental systems and methods used in many studies. Here, indicate whether each material, system or method listed is relevant to your study. If you are not sure if a list item applies to your research, read the appropriate section before selecting a response.

Materials & experimental systems

n/a	Involved in the study	
☒	☐	Antibodies
☒	☐	Eukaryotic cell lines
☒	☐	Palaeontology and archaeology
☒	☐	Animals and other organisms
☐	☒	Human research participants
☒	☐	Clinical data
☒	☐	Dual use research of concern

Methods

n/a	Involved in the study	
☒	☐	ChIP-seq
☒	☐	Flow cytometry
☒	☐	MRI-based neuroimaging

Human research participants

Policy information about studies involving human research participants

Population characteristics	The human participants in this study are elder female patients underwent breast cancer removal or breast reduction. The exact age and race of each participant are not available to the investigators.
Recruitment	Subjects at Carle Foundation Hospital, Urbana, IL preoperatively received our research protocol, knowing the purpose and content of our work, and provided written informed consent permitting the investigational use of their tissue. Additional human breast tissue specimens were obtained from the Cooperative Human Tissue Network (CHTN), established in 1987 by the National Cancer Institute in response to an increase in the demand for high quality biospecimens for cancer research.
Ethics oversight	Institutional Review Boards at the University of Illinois at Urbana-Champaign and Carle Foundation Hospital, Urbana, IL.

Note that full information on the approval of the study protocol must also be provided in the manuscript.
